# Skeletal muscle fat quantification by dual-energy computed tomography in comparison with 3T MR imaging

**DOI:** 10.1007/s00330-021-07820-1

**Published:** 2021-03-26

**Authors:** I. Molwitz, M. Leiderer, R. McDonough, R. Fischer, A-K. Ozga, C. Ozden, E. Tahir, D. Koehler, G. Adam, J. Yamamura

**Affiliations:** 1 Departement of Diagnostic and Interventional Radiology and Nuclear Medicine, University Medical Center Hamburg-Eppendorf, Martinistraße 52, 20246 Hamburg, Germany; 2 Department of Diagnostic and Interventional Neuroradiology, University Medical Center Hamburg-Eppendorf, Martinistraße 52, 20246 Hamburg, Germany; 3 UCSF Benioff Children’s Hospital Oakland, Oakland, CA USA; 4 Institute of Medical Biometry and Epidemiology, University Medical Center Hamburg-Eppendorf, Martinistraße 52, 20246 Hamburg, Germany

**Keywords:** Tomography, spiral computed, Sarcopenia, Muscles

## Abstract

**Objectives:**

To quantify the proportion of fat within the skeletal muscle as a measure of muscle quality using dual-energy CT (DECT) and to validate this methodology with MRI.

**Methods:**

Twenty-one patients with abdominal contrast-enhanced DECT scans (100 kV/Sn 150 kV) underwent abdominal 3-T MRI. The fat fraction (DECT-FF), determined by material decomposition, and HU values on virtual non-contrast-enhanced (VNC) DECT images were measured in 126 regions of interest (≥ 6 cm^2^) within the posterior paraspinal muscle. For validation, the MR-based fat fraction (MR-FF) was assessed by chemical shift relaxometry. Patients were categorized into groups of high or low skeletal muscle mean radiation attenuation (SMRA) and classified as either sarcopenic or non-sarcopenic, according to the skeletal muscle index (SMI) and cut-off values from non-contrast-enhanced single-energy CT. Spearman’s and intraclass correlation, Bland-Altman analysis, and mixed linear models were employed.

**Results:**

The correlation was excellent between DECT-FF and MR-FF (*r* = 0.91), DECT VNC HU and MR-FF (*r* = - 0.90), and DECT-FF and DECT VNC HU (*r* = − 0.98). Intraclass correlation between DECT-FF and MR-FF was good (*r* = 0.83 [95% CI 0.71–0.90]), with a mean difference of - 0.15% (SD 3.32 [95% CI 6.35 to − 6.66]). Categorization using the SMRA yielded an eightfold difference in DECT VNC HU values between both groups (5 HU [95% CI 23–11], 42 HU [95% CI 33–56], *p* = 0.05). No significant relationship between DECT-FF and SMI-based classifications was observed.

**Conclusions:**

Fat quantification within the skeletal muscle using DECT is both feasible and reliable. DECT muscle analysis offers a new approach to determine muscle quality, which is important for the diagnosis and therapeutic monitoring of sarcopenia, as a comorbidity associated with poor clinical outcome.

**Key Points:**

*• Dual-energy CT (DECT) material decomposition and virtual non-contrast-enhanced DECT HU values assess muscle fat reliably.*

*• Virtual non-contrast-enhanced dual-energy CT HU values allow to differentiate between high and low native skeletal muscle mean radiation attenuation in contrast-enhanced DECT scans.*

*• Measuring muscle fat by dual-energy computed tomography is a new approach for the determination of muscle quality, an important parameter for the diagnostic confirmation of sarcopenia as a comorbidity associated with poor clinical outcome.*

## Introduction

Sarcopenia is associated with a lower life expectancy [1, 2] and poorer prognosis in cancer patients [3]. It is also associated with increased rates of complication in patients requiring surgery or following trauma [4] and leads to longer hospitalization stays [4, 5]. Detecting sarcopenia is thus vital to the planning and initiation of appropriate nutrition and exercise regimes.

The current most widely cited definition [6] from the recently updated European consensus of the European Working Group on Sarcopenia in Older People states that sarcopenia is probable in individuals with low muscle strength and recommends diagnosis confirmation by the detection of low muscle quantity and quality [7].

While muscle strength can be measured clinically e.g. by the handgrip test, muscle quantity can be assessed by bioelectrical impedance analysis (BIA), dual-energy absorptiometry (DXA), computed tomography (CT), or magnetic resonance imaging (MRI) [7]. BIA values, however, are influenced by the patient’s hydration status while DXA relies on certain assumptions regarding the distribution of muscle and fat compartments due to its two-dimensional nature [8]. CT and MRI, conversely, accurately allow the determination of the body composition [9, 10]. CT is especially advantageous in patients who already may require CT examinations for clinical indications, as is frequently the case in chronically and/or severely ill patients [6]. Although less routinely used in primary care, CT and MRI are considered to be the gold standard for the determination of muscle quantity [7, 11].

To date, however, there is no consensus on how or with which technique muscle quality, as a measure of muscle strength per unit size [12] should be assessed [7]. An increase in intra- and extramyocellular fat has been shown to be indicative of a reduction in muscle quality [12]. Thus, studies on the ability of radiological descriptions of CT images or skeletal muscle mean radiation attenuation (SMRA) to assess the fat accumulation of the skeletal muscle have been performed [13]. While the former represents a subjective method, the determination of SMRA is semiquantitative, with values ranging between - 190 and +150 Hounsfield units (HU) and a peak at 50 HU [14]. Moreover, SMRA is influenced by the use of iodinated contrast agents [13]. Information regarding the use of such agents and phase acquisition are often missing in non-radiological papers on the clinical impact of SMRA values [13], which complicates comparability between studies.

On the contrary, dual-energy CT (DECT) scanners, which have become increasingly more common in the clinical routine, offer new, quantitative, and contrast agent-independent approaches for measuring fat. Dual-energy is characterized by the generation of two different energy spectra, which can be derived from two independent tube detector systems as in dual-source DECT [15]. With DECT, the proportion of a certain material or tissue within a voxel, e.g., fat, can be determined by the energy-dependent material-specific attenuation coefficients [16]. Furthermore, virtual non-contrast-enhanced (VNC) images can be created from DECT scans which were performed with contrast medium [15]. The quantification of fat by DECT material decomposition or by employing the radiation attenuation values on VNC images has been successfully demonstrated for the liver, the bone marrow, and the adrenal glands [17].

The major purpose of this pilot study was to quantify fat within the skeletal muscle as a measure of muscle quality by DECT material decomposition and DECT VNC HU values in comparison to MR chemical shift relaxometry (MRCSR). Furthermore, an association between DECT values and sarcopenia classifications based on SMRA cut-off values from non-contrast-enhanced single-energy CT scans and single-energy CT-derived parameters of muscle quantity was investigated. To the best of our knowledge, this is the first study that applies DECT fat quantification to the skeletal muscle.

## Materials and methods

### Study population

For this prospective study, 22 Caucasian patients were consecutively recruited according to the *a priori* inclusion criterion of a contrast-enhanced standardized abdominal dual-source DECT scan (SOMATOM Force®, Siemens). The exclusion criteria included contraindications for MRI examination, such as claustrophobia, foreign bodies (e.g., pacemakers) that were not at least MRI conditional at 3T, and patients who were unable to provide consent. Care was taken to equally include patients of either sex and of varying ages and body mass indices (BMI). Informed and written consent was obtained from all participants. All patients received a subsequent abdominal 3-T MRI scan (Ingenia®, Philips Healthcare) without contrast medium after a median time interval of 2 days. One patient prematurely terminated the MR examination due to abdominal pain. The final patient collective thus consisted of 21 Caucasian patients (Table 1). Study protocols and procedures were conducted in compliance with the Declaration of Helsinki and in accordance with local ethical guidelines; the local ethics committee approved the study.

**Table 1:** Study population*.* Overview of gender, age, size, weight, body mass index, and primary condition of all included patients. The interval between dual-energy computed tomography (CT) and magnetic resonance imaging scan in days, as well as the clinical CT indication, is provided

Patient	Gender	Age	Height [m]	Weight [kg]	Body mass index [kg/m^2^]	Time interval CT and MR [d]	Primary condition	CT indication
1	f	60	1.68	82	29.1	2	Arterial hypertension	Bleeding?
2	f	33	1.61	85	32.8	4	Post-partum (caesarean)	Infected hematoma?
3	m	80	1.73	78	26.1	4	Hemiparesis SP stroke, COPD, DM type II, alcohol abuse	Diverticulitis? perforation?
4	f	53	1.77	67	21.4	4	Pancreas carcinoma (pT2, N2 M0)	Current staging
5	f	81	1.62	85	32.4	3	PAD stage IV, COPD, DM type II, alcohol abuse, CKD stage IV, steatosis hepatis	SP aortic prosthesis removal
6	m	77	1.80	75	23.2	2	Hemiparesis, NYHA III, COPD D, SCLC (pT1c, N0, M0), CLL	Acute abdomen
7	f	55	1.67	71,2	25.5	2	Malignant melanoma (pT2b, N2c, M1c), bedridden (epidural metastases), hepatic steatosis	Bowel obstruction?
8	m	65	1.76	53	17.1	1	NSCLC (cT4, cN3, cM1c), SP NSTEMI, SP hypoxic encephalopathy	Focus of infection? abscess? GI dysfunction?
9	f	58	1.83	72	21.5	4	Aggressive fibromatosis	Ulcus perforation? pancreatitis?
10	m	52	1.86	103	29.8	3	DM, Hypercholesterolemia, steatosis hepatis	Pancreatitis? abdominal focus?
11	m	37	1.94	88	23.4	2	Glioblastoma stage IV	Focus of infection?
12	f	58	1.56	43	17.7	1	Non-Hodgkin lymphoma relapse (Ann-Arbor IV), HIV	Focus of infection? abscess?
13	f	68	1.6	72	28.1	1	Breast cancer (pT2, pN2a, M1), SP intestinal perforation	Cholestasis? abscess?
14	f	83	1.63	67	25.2	4	Klatskin tumor type IIIa/IV	Perfusion deficit? portal vein thrombosis? cholestasis?
15	m	62	1.78	109	34.4	2	Prostate cancer (pT1b, N0, M0), SP ileum perforation, hepatic steatosis	Focus of infection?
16	m	51	1.79	89	27.8	2	Chronic hepatitis C, liver cirrhosis, AKI	Portal vein thrombosis? hepatic decompensation?
17	m	27	1.78	55	17.4	36	SP multiples abscesses, SP partial lung resection, DM type I	Abscess?
18	m	42	1.81	60,5	18.5	3	PSC, cirrhosis, TIPS	Focus of infection?
19	m	60	1.83	68	20.3	4	SP papilloma resection	GI dysfunction? pancreatitis?
20	m	32	1.81	73	22.3	3	-	Appendicitis?
21	f	57	1.71	72	24.6	1	Colorectal cancer (pT3, N2b, M1a)	Progress? infection?

*Abbreviations*: *SP*, status post; *COPD*, chronic obstructive pulmonary disease; *DM*, diabetes mellitus; *PAD*, peripheral artery disease; *CKD*, chronic kidney disease; *CLL*, chronic lymphocytic leukemia; *AKI*, acute kidney injury; *PSC*, primary sclerosing cholangitis; *TIPS*, transjugular intrahepatic portosystemic shunt

### DECT scan and post-processing

**Fig. 1:**
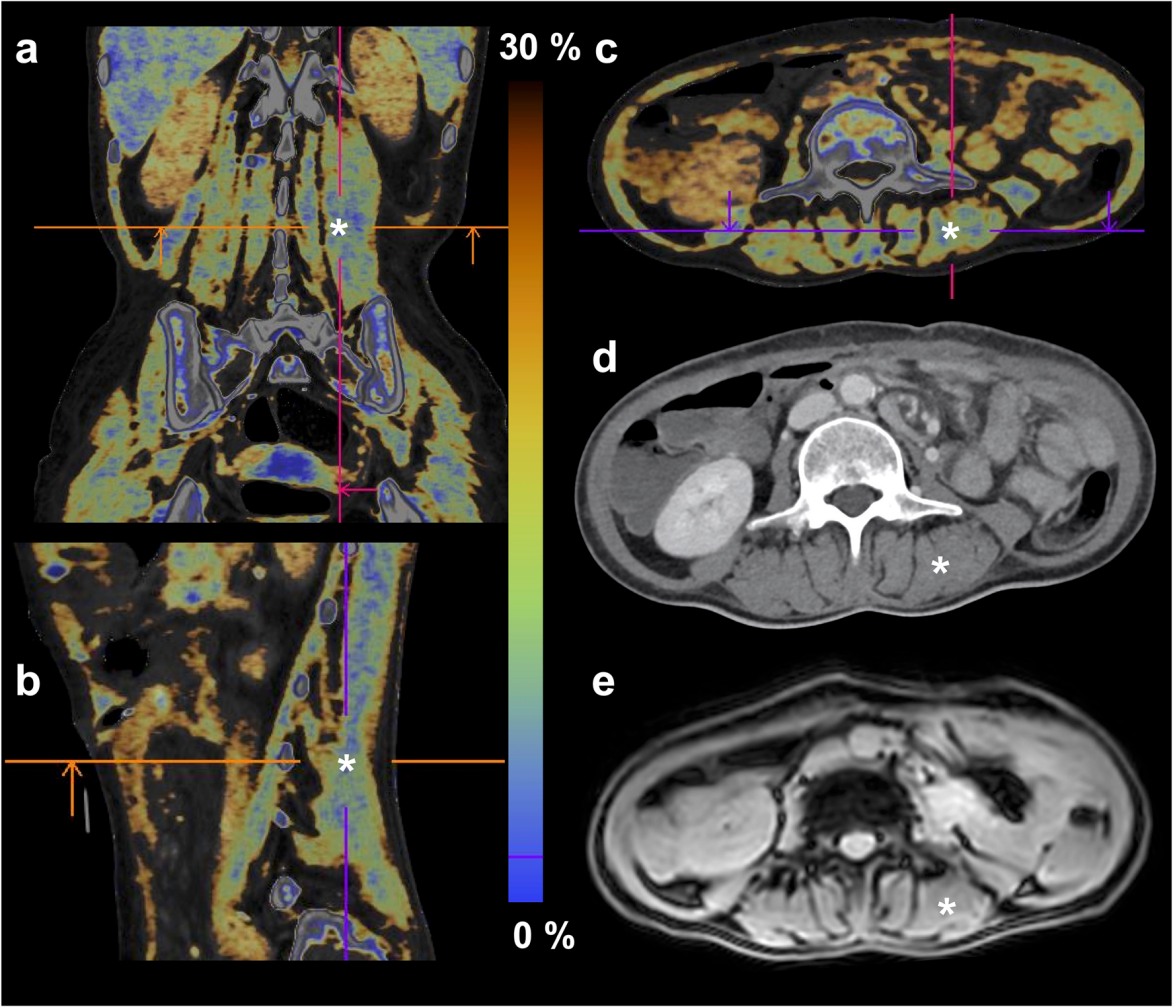
Fat map images (**a**–**c**) calculated from dual-energy computed tomography (**d**), and magnetic resonance magnitude image (**e**) of a 58-year-old female patient with non-Hodgkin lymphoma. The analyzed region of the right posterior paraspinal muscle is marked (white asterisk, cross-hairs on fat maps)

DECT parameters of the standard abdominal protocol were 100 kV/Sn 150 kV, pitch 0.5, collimation 0.6 mm, slice thickness 1 mm (reconstructed 5 mm). Pixel size was 0.6 × 1 mm. Image acquisition started 80 s after injection of 80 ml Iomeprol as a contrast agent (Imeron 350 M, Bracco IMAGING). As a first step, VNC images were created with the commercially available SyngoVia software (Liver VNC, Siemens). The algorithm is based on a three-material decomposition, assuming each voxel consists of liver tissue, fat, and iodine. Subsequently, the Liver Fat Map (Siemens) application, which shows the calculated fat concentration as a color-coded map (Fig. 1a–c), was applied to the whole data set, including the skeletal muscle.

**Fig. 2:**
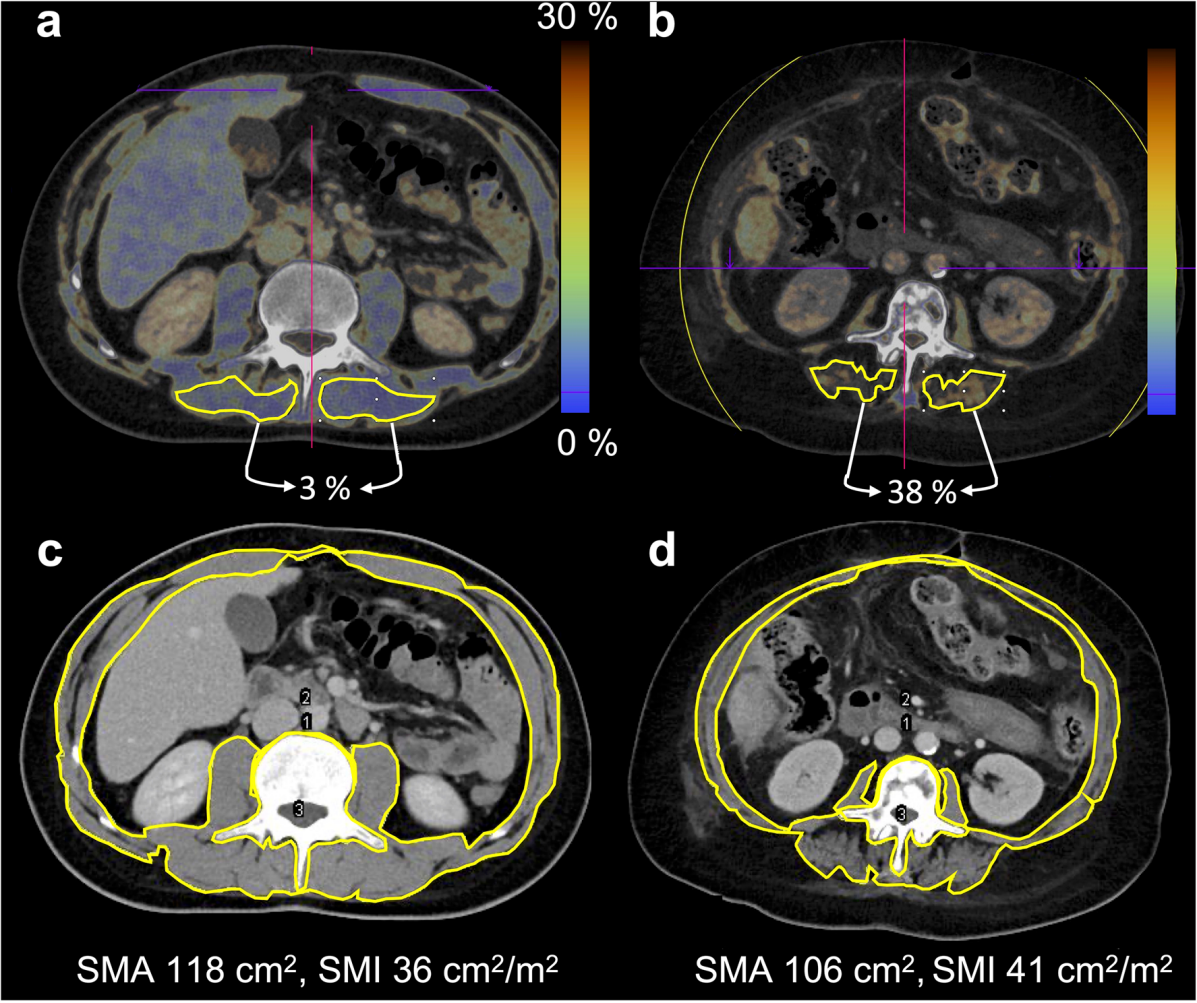
Determination of DECT fat fraction, DECT VNC HU, and SMA. Dual-energy computed tomography fat fraction (DECT-FF) and DECT virtual non-contrast-enhanced Hounsfield units (DECT VNC HU) were acquired from regions of interest (ROIs) of the posterior paraspinal muscles (m. erector spinae) (**a**, **b**). The skeletal muscle area (SMA) was determined after delimitation of muscle-specific tissue (−29 to +150 HU) by subtraction of the perimeter of the third lumbar vertebra and the inner skeletal muscle perimeter from the outer skeletal muscle perimeter [18] (**c**, **d**). The skeletal muscle index (SMI) was derived by SMA/height^2^. Two patients with low muscle fat percentage of 3% in DECT-FF and low SMI (**a**, **c**: 32 y, m, no pre-existing conditions) and high muscle fat of 38% in DECT-FF and higher SMI (**b**, **d**: 68 y, f, metastasized breast cancer) are exemplarily shown

Regions of interest (ROIs) were defined on axial slices at the height of the third lumbar vertebra (LV3) (Fig. 1, marked by a white asterisk), as this is also the standard height for the determination of muscle quantity in single-energy CT [3, 13] and drawn along the inner borders of the left and right posterior paraspinal muscles (m. erector spinae) (Fig. 2a, b). Three consecutive slices were analyzed to compensate for potential fluctuations within the muscle. Each ROI was at least 6 cm^2^. The DECT fat fraction (DECT-FF), as well as DECT VNC HU, was noted for each ROI and averaged on each side of the spine. All ROIs were independently defined by two radiologists (4 and 2 years of training) to determine the interobserver reliability.

To also assess muscle quantity, images at the LV3 were exported as DICOM files and further processed with the open-source software Image J (National Institutes of Health, Laboratory for Optical and Computation Instrumentation). Outer and inner perimeters of the abdominal muscles, as well as the outer perimeter of the LV3, were contoured (Fig. 2c, d). After application of a threshold of - 29 to +150 HU to demarcate intermuscular adipose tissue [14, 18], the skeletal muscle area (SMA) which, at this height, has been shown to be highly correlated with the total body skeletal muscle mass [19], was calculated according to Gomez-Perez et al [18].

### MR imaging and post-processing

MRI sequences included a coronal (TR 875 ms) and sagittal T2-weighted sequence (TR 528 ms, TE 80 ms, FA 90°) for anatomical information. A transverse 3D gradient echo sequence with 20 echoes (1^st^ TE 1.2 ms, ∆TE 1.1 ms, TR 24 ms, FA 3°) was applied for MRCSR. Voxel size of gradient echo sequences was 1.5 × 1.5 × 4 mm.

ROIs (≥ 6 cm^2^) were drawn on the magnitude images (Fig. 1e) in accordance with the DECT images. An in-house fitting algorithm [20], based on the lipid spectral model of Hamilton et al [21] for the liver, was used to determine the MR fat fraction (MR-FF) from the magnitude images of the first 7 echoes [22]. The fitting algorithm was adapted to muscle tissue by providing the fat fraction originating from the main lipid peaks at 1.3 ppm (434 Hz at 3T; intramyocellular lipid) and 1.5 ppm (409 Hz; extramyocellular lipid).

### Sarcopenia classification

Based on the SMA values, the skeletal muscle index (SMI) was calculated using SMA [cm^2^]/height [m]^2^ [23]. The SMI as a parameter of muscle quantity was used to classify patients as sarcopenic or non-sarcopenic, according to three different cut-off-based systems: (1) Prado et al, who classify SMI according to gender [24]; (2) Martin et al, who, for male patients, also take BMI into consideration [25]; and (3) van der Werf et al, who classify SMI according to gender, BMI, and age [26] (Table 2).

**Table 2:** Patient classification as sarcopenic (+) and non-sarcopenic (-), according to different indices*.* Patients were assigned to either a sarcopenic ((+) skeletal muscle index (SMI) below cut-off) or non-sarcopenic ((-) SMI above cut-off) group, according to the SMI-based classification systems of Prado et al (sarcopenia: men ≤ 52.4 cm^*2*^/m^*2*^; women ≤ 38.5 cm^*2*^/m^*2*^), Martin et al (sarcopenia: men with BMI < 25 and SMI < 43 cm^*2*^/m^*2*^ or with BMI ≥ 25 and SMI < 53 cm^*2*^/m^*2*^, women BMI independent < 41 cm^*2*^/m^*2*^), and van der Werf et al (below the 5^th^ gender-, age-, and BMI-specific percentile of a healthy Caucasian population). By using a cut-off value for skeletal muscle mean radiation attenuation (SMRA) from non-contrast-enhanced single-energy CT scans of 29 HU, they were furthermore categorized into groups of high or low SMRA by the HU values of the left and right posterior paraspinal muscle on DECT virtual non-contrast-enhanced images

Patient	SMI [cm^2^/m^2^]	SMRA left posterior paraspinal muscle [HU]	SMRA right posterior paraspinal muscle [HU]	Sarcopenia classification results (+ sarcopenic, - non-sarcopenic)				
				Prado et al	Martin et al	van der Werf et al	SMRA < 29 HU	
							left	right
1	33.47	42	39	+	+	-	-	-
2	48.61	45	43	-	-	-	-	-
3	37.86	32	27	+	+	+	-	+
4	34.60	46	44	+	+	-	-	-
5	49.54	35	34	-	-	-	-	-
6	37.21	38	40	+	+	-	-	-
7	28.94	41	45	+	+	+	-	-
8	28.24	35	33	+	+	+	-	-
9	38.74	38	38	-	+	-	-	-
10	43.65	53	55	+	+	+	-	-
11	31.66	57	53	+	+	+	-	-
12	29.95	37	35	+	+	-	-	-
13	35.55	2	-3	+	+	-	+	+
14	32.67	29	25	+	+	-	+	+
15	54.20	60	62	-	-	-	-	-
16	46.46	30	37	+	+	-	-	-
17	32.61	55	55	+	+	+	-	-
18	29.16	55	55	+	+	+	-	-
19	45.17	50	49	+	+	-	-	-
20	32.31	53	52	+	+	+	-	-
21	32.67	29	30	+	+	-	-	-
Mean (SD) f	36.47 ± 7.19	34 ±13	33 ± 14					
Mean (SD) m	38.05 ± 8.32	47 ±11	47 ± 11					

*Abbreviations*: *SMI*, skeletal muscle index; *SMRA*, skeletal muscle mean radiation attenuation; *BMI*, body mass index; *HU*, Hounsfield unit; *DECT*, dual-energy computed tomography; *SD*, standard deviation; *f*, female; *m*, male

Patients were also separated into a group with low and high muscle quality according to the HU values on DECT VNC images as a surrogate of the SMRA. Because SMRA in non-contrast-enhanced single-energy CT is commonly judged as being low for attenuation values of 29 HU or less [13], this was used as a cut-off value (Table 2). The mean DECT VNC HU values in the group of patients below and above this cut-off were calculated and compared.

### Statistics

**Fig. 3:**
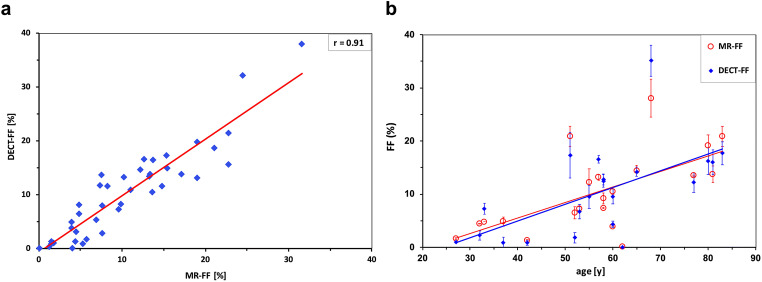
Correlation between DECT-FF and MR-FF and distribution of both plotted against patient age. Correlation of dual-energy computed tomography fat fraction (DECT-FF, blue) and fat fraction from magnetic resonance chemical shift relaxometry (MR-FF, red) was high (r = 0.91) (**a**). A higher patient age appears to be moderately correlated with higher DECT-FF (r = 0.62) and MR-FF (r = 0.59) (**b**)

Patient characteristics were summarized with descriptive statistics. Because of the skewed distribution of measurements, Spearman’s correlation was used to determine the correlation between MR-FF and DECT-FF (Fig. 3a), MR-FF and DECT VNC HU, DECT-FF and DECT VNC HU, as well as for these MR and DECT-derived values and patient age (Fig. 3b) or patient BMI (Table 3).

**Table 3:** Correlation between age, BMI, and imaging parameters.

Spearman’s correlation			
Parameters		r_S_	*p* value
Patient age	DECT-FF	0.62	< 0.01
	DECT VNC HU	− 0.63	< 0.01
	MR-FF	0.59	< 0.01
BMI	DECT-FF	0.22	0.17
	DECT VNC HU	0.12	0.45
	MR-FF	− 0.08	0.61
MR-FF	DECT-FF	0.91	< 0.01
	DECT VNC HU	− 0.90	< 0.01
DECT VNC HU	DECT-FF	− 0.98	< 0.01
SMA	MR-FF	− 0.31	0.05
	DECT-FF	− 0.35	0.02
	DECT VNC HU	0.35	0.02

*Abbreviations*: *DECT-FF*, dual-energy computed tomography fat fraction; *DECT VNC HU*, DECT virtual non-contrast-enhanced Hounsfield units; *MR-FF*, magnetic resonance fat fraction; *BMI*, body mass index; *SMA*, skeletal muscle area

**Fig. 4:**
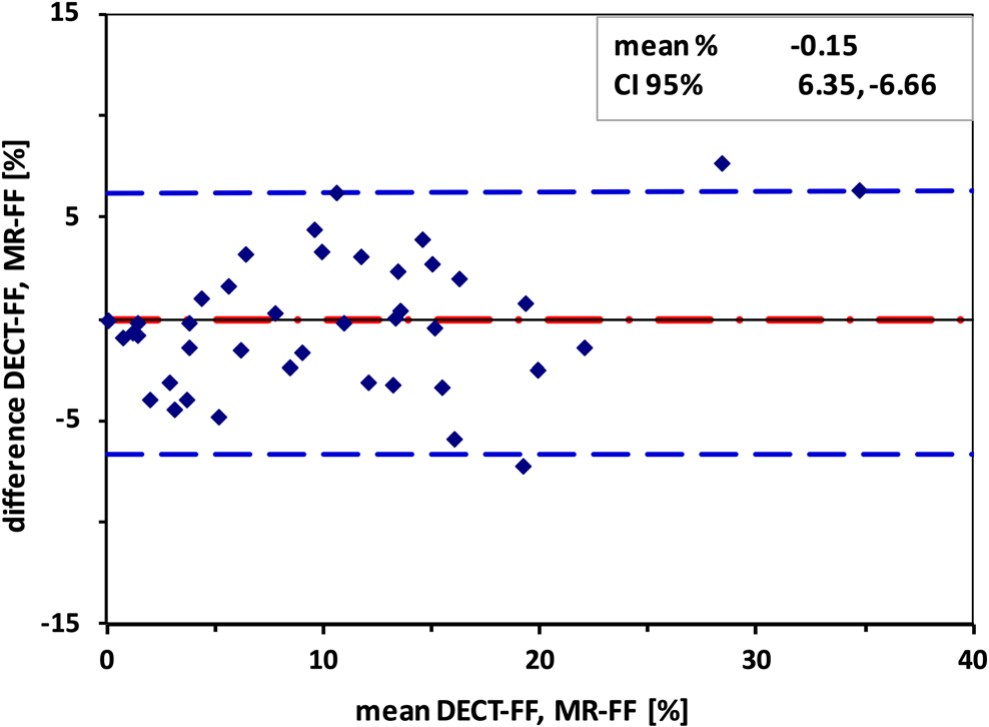
Bland-Altman plot of DECT-FF and MR-FF. Mean difference between dual-energy computed tomography fat fraction (DECT-FF) and magnetic resonance chemical shift relaxometry fat fraction (MR-FF) was *−*0.15%. 95% confidence interval (95% CI) was approximately 6.5%. The highest difference of 7.74% was found for the patient with the highest fat fraction within the study population (38% in DECT and 32% in MR, within the right posterior paraspinal muscles)

The agreement between DECT-FF and MR-FF was tested with the intraclass correlation coefficient and the mean difference calculated by the Bland-Altman analysis (Fig. 4). For interobserver reliability of both DECT-FF and DECT VNC HU, Cohen’s kappa was calculated.

A mixed linear model was used to test the relationship between log-transformed DECT-FF and DECT VNC HU to the three SMI-based sarcopenia classifications and between log-transformed DECT VNC HU and the SMRA-based categorization. DECT-FF and DECT VNC HU were log-transformed as they had a skewed distribution. This resulted in approximately normally distributed data thus allowing for the application of a linear model. To account for the two measurements for DECT-FF and DECT VNC HU per individual (one per side = 2 for each individual), a mixed model approach was used. Variance was additionally explained with an extra random term for each side of the patient. As all analyses were explorative in nature, *p* values are considered descriptive.

## Results

### Study population

There was equal representation of male (*n* = 11, 52%) and female (*n* = 10, 48%) patients within the collective (Table 1). Age ranged from 27 to 83 years, with a median of 58 years. The median BMI was 24.6 and ranged from 17.1 to 34.4 kg/m^2^. The major primary condition was cancer of varying origin (*n* = 11, 52%). The most common CT indication was search for a focus of infection or abscess (*n* = 8%) (Table 1).

The mean skeletal muscle fat fraction was 10.22% (median 10.70%) in DECT-FF and 10.37% (median 8.90%) in MR-FF. The mean and median DECT VNC HU values were both 41 HU. In one patient, no muscle fat could be detected; this also corresponded to the highest measured DECT VNC HU values of 60 and 62 HU on either side of the posterior paraspinal muscles. One patient showed exceptionally high fat fractions, with 37.99% in DECT and 31.60% in MRI (Fig. 3). The maximum difference of the fat fraction between both sides of the posterior paraspinal muscle within the same patient was 8.35% by DECT-FF and 7.10% by MR-FF. The mean difference between muscles of either side within the same patients was 2.51% by DECT-FF and 1.86% by MR-FF.

### Correlation and agreement

Spearman’s correlation was excellent between MR-FF and DECT-FF (*r* = 0.91) (Fig. 3a), MR-FF and DECT VNC HU (*r* = − 0.90), and DECT-FF and DECT VNC HU (*r* = − 0.98).

Correlation between patient age and the varying image parameters was moderate: MR-FF (*r* = 0.59) (Fig. 3b), DECT-FF (*r* = 0.62) (Fig. 3b), and DECT VNC HU (*r* = − 0.63) (Table 3). BMI was observed to be poorly correlated with MR-FF (− 0.08), DECT-FF (*r* = 0.22), and DECT VNC HU (0.12), while little correlation was found between SMA and MR-FF (*r* = − 0.31), SMA and DECT-FF (*r* = − 0.35), or SMA and DECT VNC HU (*r* = 0.35) (Table 3).

Intraclass correlation between log-transformed DECT-FF and MR-FF was good (*r* = 0.83, CI 95% 0.71–0.90). The Bland-Altman analysis yielded a mean difference of - 0.15% (SD 3.32, CI 95% 6.35 to - 6.66) (Fig. 4). Interobserver reliability (Cronbach’s alpha) was 0.99 for DECT-FF and 0.98 for DECT VNC HU.

### DECT values and sarcopenia indices

According to Prado et al, 17 of 21 patients would have been classified as sarcopenic, 18 according to Martin et al, and 8 according to van der Werf et al (Table 2).

However, the mixed linear model demonstrated no clear relationship between the DECT-derived values (DECT-FF, DECT VNC HU) and the named sarcopenia classifications (Table 4). Indeed, the calculated mean of the DECT-FF of all patients who would have been categorized as sarcopenic by van der Werf et al was 2.5% while it was 8.2% for patients who would have been classified as non-sarcopenic (*p = *0.013). Correspondingly, DECT VNC HU values were higher in patients classified as sarcopenic by van der Werf et al, with 46 HU compared to 29 HU in non-sarcopenic patients (*p* = 0.136). While in patients categorized as sarcopenic by Prado et al and Martin et al as anticipated mean MR-FF and mean DECT-FF were consistently higher and DECT VNC HU lower in sarcopenic than in non-sarcopenic patients, those differences were only significant for MR-FF (Table 4).

**Table 4:** Relationship between MR-FF, DECT-FF, DECT VNC HU, and SMI classification systems according to the mixed linear model. Patients classified as sarcopenic by Prado et al and Martin et al showed higher MR and DECT skeletal muscle fat fractions (MR-FF, DECT-FF) and lower DECT VNC HU values than non-sarcopenic patients. However, the 95% confidence intervals (95% CI) were large, and results were only significant for MR-FF. Patients who would have been classified as sarcopenic by van der Werf et al showed lower DECT-FF and MR-FF and higher DECT VNC HU values than patients which would have been classified as non-sarcopenic

	Sarcopenia classification	Sarcopenic [95% CI]	Non-sarcopenic [95% CI]	*p* value
MR-FF	Prado et al	8.33 [5.55–12.52]	2.78 [1.20–6.44]	0.022
	Martin et al	8.37 [5.71–12.27]	1.87 [0.73–4.77]	0.005
	van der Werf et al	5.60 [2.98–10.51]	7.59 [4.64–12.44]	0.445
DECT-FF	Prado et al	5.80 [3.38–9.92]	3.35 [1.07–9.86]	0.349
	Martin et al	6.03 [3.61–10.07]	2.12 [0.6–7.45]	0.128
	van der Werf et al	2.47 [1.19–5.14]	8.20 [4.61–14.57]	0.013
DECT VNC HU	Prado et al	32.79 [22.87–46.34]	43.60 [21.35–89.12]	0.472
	Martin et al	33.05 [23.59–46.29]	45.70 [20.03–104.27]	0.467
	van der Werf et al	46.39 [28.28–76.02]	28.90 [19.61–42.61]	0.136

*Abbreviations*: *DECT*, dual-energy computed tomography; *DECT VNC HU*, *DECT*, virtual non-contrast-enhanced Hounsfield units

Following the application of the SMRA cut-off for low radiodensity skeletal muscle from non-contrast-enhanced single-energy CT to the measured DECT VNC HU values, 5 measurements from 3 patients would have been classified as sarcopenic (Table 2). The mean DECT VNC HU in the group below and above the SMRA cut-off was 5 HU (95% CI 2.5–10.9) and 43 HU (95% CI 32.9–56.3), respectively, an approximately eightfold difference in mean muscle HU between both groups (*p* = 0.05).

## Discussion

In this prospective study, DECT fat quantification was applied to the skeletal muscle and validated by MRCSR. The major findings were (a) DECT material decomposition algorithms originally designed for the determination of liver fat deliver valid results for the fat fraction of the skeletal muscle; (b) in contrast-enhanced DECT scans, patients can be categorized into groups of low and high SMRA using SMRA cut-off values from non-contrast-enhanced single-energy CT with the help of DECT VNC HU values; (c) no valid relationship was found between the DECT skeletal muscle fat fraction and sarcopenia classifications systems based on the SMI.

The currently available literature regarding DECT fat quantification focuses on the liver, the bone marrow, or its usefulness in the differentiation of benign and malignant lesions, e.g., in the adrenal gland [17]. As a result, the comparability of our results is limited. However, compared to the level of correlation between DECT-FF and MR-FF for rabbit (*r* = 0.65 [27]) or mice livers (*r*^*2*^ ≤ 0.67 [28]), the correlation for the skeletal muscle in this study was higher. The mean difference between DECT-FF and MR-FF in this study was lower, but showed a larger standard deviation than described for rabbit livers (1.56% ± 1.96% [27]). The correlation of DECT-FF to MR-FF in the skeletal muscle was similar to that described for patient and cadaver bone marrow in other dual-source DECT studies (*r* = 0.77 [29], *r* = 0.88 [30]). Despite study heterogeneity and different target organs, we thus conclude that DECT material decomposition is appropriate for fat quantification, not only within the liver and the bone marrow but also in the skeletal muscle.

Likewise, the level of correlation was high between DECT VNC HU and MR-FF for the skeletal muscle in this study and between non-contrast-enhanced DECT HU values at 65 keV and MR-FF in mice liver (*r*^*2*^ = 0.86 [28]). This also demonstrates a major advantage of DECT: the possibility to create VNC images from a contrast-enhanced examination [15] and perform HU analyses on these. The fact that VNC and real non-contrast-enhanced HU values in the muscles are either not significantly different or show a difference of less than 2 HU in absolute numbers has been previously demonstrated [31–33]. Sarcopenia cut-off values for SMRA from non-contrast-enhanced single-energy CT scans should thus hypothetically be applicable to VNC images of contrast-enhanced DECT scans. While the mean DECT VNC HU values differed greatly between the patient groups below and above the SMRA cut-off value from non-contrast-enhanced single-energy CT in our study, the absolute numbers were not sufficient to validly confirm this hypothesis. Nevertheless, the potential to classify patients as sarcopenic by their DECT VNC HU values and established SMRA cut-off values from non-contrast-enhanced single-energy CT is of high clinical relevance, as the majority of indicated examinations in severely and/or chronically ill patients requires the use of a contrast agent. Additional pre-contrast scanning, which was commonly applied in abdominal CT imaging in the past, is no longer recommended [34] as for most indications, it does not provide further diagnostic information but increases radiation dose significantly [35, 36]. A method to assess SMRA independent of the use and phase of contrast agent or other contrast independent approaches for muscle fat quantification as DECT material decomposition is therefore highly desirable.

Interestingly, higher muscle fat means were found in non-sarcopenic patients compared to sarcopenic patients classified according to the SMI-based classification system of van der Werf et al while differences in DECT values were not significant between sarcopenic and non-sarcopenic patients according to Prado and Martin et al. There are likely several explanations for these observations: first, the SMI-based classification systems might not always be suited to accurately assess sarcopenia and thus be predictive of worse outcomes in sarcopenic patients. This is supported by a review article on the predictive value of different SMI cut-off values for gastrointestinal cancer [37]. Here, an overall association between SMI classification and poor perioperative outcome was found but a lack of predictive value for the cut-off of Martin et al regarding major complications was also described [37]. Furthermore, while sarcopenia, as assessed by Martin et al, appeared to influence overall mortality, this could not be demonstrated for Prado et al [37]. Study results for different cancer entities are also discrepant; e.g., muscle quantity was shown to be a predictive parameter for survival in pancreatic cancer [38] but not for esophageal cancer [39]. The literature on the classification system of van der Werf et al which was first published in 2018 [26] is rare and no large reviews on its ability to accurately assess sarcopenia exist, yet.

Secondly, the SMI does not take the intra- and extramyocellular fat content into consideration. After application of a threshold of - 29 and +150 HU to delimitate fat from muscle tissue, the measured SMA can be low, while the extra- and intramyocellular fat content within the muscle fascia is high [40]. The SMA would however be comparably low if no accumulation of fat tissue occurred, e.g., due to a catabolic metabolism in cachexic patients [41]. The different metabolic states are thus not reflected by the SMI.

Regardless, it appears that the SMA-based classification systems of muscle quantity alone may not be sufficient and do not necessarily agree with the muscle fat fraction as a parameter of muscle quality. New parameters to assess muscle quality are expected to gain importance [7], highlighting the potential of DECT fat quantification in the skeletal muscle. The fact that classification results differ between the cut-off systems within one patient (Table 2) moreover demonstrates that all parameters of muscle quantity or quality are probably rather part of a continuum with sarcopenic and non-sarcopenic patients at both ends and—despite being the common approach—cannot be separated by strict cut-off values.

The main limitations of this pilot study are the low patient number and the heterogeneity of the patient collective. As it was the purpose to investigate agreement of DECT and MRI fat quantification in the skeletal muscle independent of the individual’s muscle status, patients of varying age and BMI were included and the inclusion or exclusion criteria not restricted to specific primary conditions or treatments, e.g., cancer type and stage, metabolic diseases such as diabetes, status post-trauma or post-surgery, chemotherapy or treatment with steroids, all of which can influence the muscle status. However, the high variation of the skeletal muscle fat percentage resulting from the heterogenous patient collective and a broad range of BMI values should be considered another possible cause for the observed lack of agreement between the muscle fat fraction and the SMI-based sarcopenia classifications, as the latter do not take the intra and extramyocellular fat into account. Also, due to the missing agreement between DECT and SMI classification results, as well as the small number of observations in the group below the SMRA cut-off value, no conclusions on the suitability of DECT muscle parameters to determine sarcopenia, and thus, clinical outcome can be drawn.

In contrast to most single-energy CT studies on the SMRA [13], the ROIs for the SMRA, MR-FF, and DECT-FF were determined without delimitation of muscle tissue by radiodensity ranges because the fat map tool also did not include such preselection. MRCSR was chosen as the modality of comparison as opposed to the alternative reference standard MR spectroscopy (MRS); it is fast, robust, and allows for the measurement of fat content over large ROIs, which is advantageous in individuals with inhomogeneous muscle fat infiltration [42]. MRS and MRCSR have been shown to be well correlated [43].

Prospectively, studies with larger, more homogeneous patient populations should be carried out to validate the applicability of the non-contrast-enhanced single-energy SMRA cut-off to identify sarcopenic patients by their DECT VNC values and to determine gender and age-specific DECT muscle fat fraction thresholds for the diagnosis of sarcopenia. In this context, the expected association of the muscular fat percentage with clinical outcome in different patient cohorts, e.g., dependent on (histological) cancer type and therapy, also needs further validation. Care should be taken to differentiate between cachexia and sarcopenia as two commonly confused, yet distinct entities [44]. The assessment not only of muscle quantity and quality but also of body fat mass (as previously described [18]) is therefore advisable.

In conclusion, this pilot study demonstrates that the quantification of fat accumulation within the skeletal muscle as a parameter of muscle quality by DECT material decomposition is feasible and reliable. Furthermore, DECT VNC HU values allow evaluation of SMRA independent of the use of a contrast agent, which is beneficial if no pre-contrast scanning is applied.

DECT thus presents a new approach for the measurement of muscle quality, both quantitatively and objectively, in routine clinical CT scans. This has a high potential for the improvement of the radiological confirmation of sarcopenia as a common comorbidity in chronically or severely ill patients, strongly associated with poor clinical outcome.

## Abbreviations

BIA: Bioelectrical impedance analysis
BMI: Body mass index
DECT: Dual-energy computed tomography
DECT-FF: DECT fat fraction
DXA: Dual-energy absorptiometry
LV3: Third lumbar vertebra
MRCSR: Magnetic resonance chemical shift relaxometry
MR-FF: Magnetic resonance fat fraction
MRS: Magnetic resonance spectroscopy
ROI: Region of interest
SMA: Skeletal muscle area
SMI: Skeletal muscle index
SMRA: Skeletal muscle mean radiation attenuation
VNC: Virtual non-contrast-enhanced

## Declarations

### Guarantor

The scientific guarantor of this publication is the last author Prof. Dr. Jin Yamamura.

### Statistics and biometry

One of the authors (Ozga A-K.) has significant statistical expertise.

### Methodology

• prospective

• experimental study

• performed at one institution
